# A narrative review of economic constructs in commonly used implementation and scale-up theories, frameworks and models

**DOI:** 10.1186/s12961-020-00633-6

**Published:** 2020-10-01

**Authors:** Brown Vicki, Tran Huong, Blake Miranda, Laws Rachel, Moodie Marj

**Affiliations:** 1 Deakin Health Economics, Institute for Health Transformation, Deakin University, Geelong, 3220 Australia; 2 Global Obesity Centre, Institute for Health Transformation, Deakin University, Geelong, 3220 Australia; 3 Institute for Physical Activity and Nutrition, Deakin University, Geelong, 3220 Australia

**Keywords:** Implementation, scale-up, theories, models, frameworks, economic

## Abstract

**Background:**

Translating research evidence into practice is challenging and, to date, there are relatively few public health interventions that have been effectively and cost-effectively implemented and delivered at scale. Theories, models and frameworks (herein termed ‘frameworks’) have been used in implementation science to describe, guide and explain implementation and scale-up. While economic constructs have been reported as both barriers and facilitators to effective implementation and scale-up of public health interventions, there is currently no published review of how economic constructs are considered within commonly used implementation and scale-up frameworks. This paper aimed to narratively review the economic constructs incorporated in commonly used implementation and scale-up frameworks.

**Methods:**

Frameworks for inclusion in the narrative review were identified from the literature and thematic content analysis was undertaken using a recursive deductive approach. Emergent key themes and sub-themes were identified and results were summarised narratively within each theme.

**Results:**

Twenty-six framework publications were included in our analysis, with wide variation between frameworks in the scope and level of detail of the economic constructs included. Four key themes emerged from the data – ‘resources’, ‘benefit’, ‘cost’ and ‘funding’. Only five frameworks incorporated all four identified key themes. Overarching lenses from which to consider key themes included ‘stakeholder perspectives’, ‘stage in the research translation process’ and ‘context’. ‘Resources’ were most frequently considered in relation to the sub-themes of ‘types of resources’ (e.g. labour, time or infrastructure) and ‘availability’ of resources, and the opportunity for ‘economies of scale’. The ‘relative advantage of interventions’ emerged as an interconnecting sub-theme between ‘cost’ and ‘benefit’. ‘Funding’ was most often considered in relation to ‘funding sources’, ‘availability’, ‘sustainability’ or ‘contextual impact’. The concept of ‘opportunity cost’ was considered in relatively few frameworks, despite being fundamental to economic theory.

**Conclusions:**

Implementation and scale-up frameworks provide a conceptual map to inform the effective and cost-effective implementation of public health interventions delivered at scale. Despite evidence of an emerging focus on the economic considerations of implementation and scale-up within some commonly used frameworks, our findings suggest that there is significant scope for further exploration of the economic constructs related to implementation and scale-up.

## Background

The goal of public health research is increasingly focused on disseminating and implementing effective and cost-effective interventions from research to practice. Achieving a good return on investment in public health intervention research requires the successful implementation of interventions at scale, with funding bodies around the world placing increasing emphasis on the translation of knowledge into policy and practice [1]. Evidence of successful scale-up of public health interventions — the process by which health interventions shown to be efficacious on a small scale or in controlled conditions are expanded under real-world conditions into broader policy and practice [2, 3] — is limited [4, 5]. This sub-optimal translation of evidence into practice has been attributed to the social-ecological differences between controlled testing environments as well as to the challenges of implementation and scale-up in inherently more complex and dynamic real-world contexts and environments [6].

Implementation theories, models and frameworks have gained popularity over the last decade, providing much needed insight to guide successful implementation and scale-up [2, 7]. While theories attempt to explain causal mechanisms, models and frameworks are more descriptive of processes or factors influencing implementation [7]. The overarching aims of the use of theories, models and frameworks (herein termed ‘frameworks’) in implementation science have been summarised as (1) to describe and/or guide the process of translating research to practice; (2) to understand and/or explain what influences implementation and scale-up outcomes; and (3) to guide evaluation design [7]. A large number of frameworks exist, with a recent study reporting the use of over 100 different examples by implementation researchers [8].

While economic evaluation can provide important information to decision-makers on the value-for-money of interventions, to date, its application within implementation science has been limited [9]. However, resource utilisation, funding and other economic constructs have been reported as both barriers and facilitators to successful dissemination, implementation and scale-up of public health interventions [2, 4, 10, 11]. For instance, Milat et al. [2] found that costing and economic modelling of intervention approaches were key factors for the successful scale-up of public health interventions. Laws et al. [4] found that a lack of ongoing funding and workforce capacity issues were key barriers to the community-wide implementation of the Infant, Feeding, Activity and Nutrition (InFANT) programme in Melbourne, Australia.

Because economic constructs such as costs, funding and resources are critical to successful intervention implementation and scale-up, it is important that we have a clear understanding of how these constructs are incorporated into commonly used implementation and scale-up frameworks. If economic constructs are not incorporated, they may be less likely to be considered by implementation scientists when describing, guiding, planning and evaluating implementation and scale-up. This may seriously impede our understanding of how economic factors may affect the outcomes of implementation and scale-up as well as our ability to explain the success (or otherwise) of implementation and scale-up efforts. To date, no review has been published examining the ways in which economic constructs related to implementation and scale-up are considered within the most commonly used implementation and scale-up frameworks.

This narrative review therefore aims to summarise the economic constructs incorporated into commonly used implementation and scale-up frameworks. Results will inform an analysis of the economic-related gaps and areas that require further exploration or elaboration within the most commonly used frameworks by implementation researchers. This information is critical for both health economists and implementation scientists, as a better understanding of the economic-related factors associated with the adoption, implementation and sustainability of interventions may improve the real-world impact of public health interventions [12]. The review also seeks to foster important cross-disciplinary dialogue among implementation researchers [7] and contributes to a larger research project that will develop a guide to more comprehensively assess the economic considerations of intervention implementation and scale-up within health economic evaluation.

## Methods

A narrative review of the economic constructs incorporated in implementation and scale-up frameworks was conducted, informed by qualitative content analysis. The definition of economic constructs was informed by key concepts and terms from the work of Raghavan on the role of economic evaluation in dissemination and implementation research [13] and published glossaries of health economics terms [14, 15]. Reporting followed the Standards for Reporting Qualitative Research [16].

### The research question

What economic constructs are incorporated into the most commonly used implementation and scale-up frameworks?

### Sample for analysis

The unit of analysis was the framework. The recently published survey by Birken et al. [8] guided the inclusion of frameworks into our study. Birken et al. [8] examined the frameworks most commonly used by implementation scientists (*n* = 223 study participants, from 12 countries) and the ways in which they were used. The study asked survey respondents to identify the theories used as part of their implementation research or practice (using an open-ended question), the ways in which they were used and the criteria used for theory selection [8]. To ensure our review reflected the most commonly used constructs, we selected the top 15 most commonly used frameworks for inclusion into our narrative review [8]. Together, these accounted for 85% of all frameworks reported by study participants [8]. Individual frameworks omitted from our analysis were reported as commonly used by less than 2% of study participants [8].

Inclusions were then cross-referenced using the study by McKay et al. [17] to ensure a relatively comprehensive set of framework inclusions for our analysis. McKay et al. [17] conducted a Delphi study to rank the most frequently used frameworks, process models and indicators for implementation and scale-up of physical activity and nutrition interventions. Any of the most commonly reported implementation and scale-up frameworks included in McKay et al. [17] but not included in the study by Birken et al. [8] were also included into our analysis. Where implementation and scale-up frameworks have developed over time, we included both the initial framework publication and the most recently published version of the framework to examine and account for differences between the original and revised versions.

### Data extraction and analysis

Papers describing the included frameworks were imported into EndNote and two reviewers (VB, HT) extracted data using a tool developed in Microsoft Excel. Extracted data included framework publication authors, publication year, aim, a brief summary, methods used for framework development, the discipline from which the framework was developed (e.g. public health, psychology), the suggested way in which the framework should be used, and the economic constructs considered in the framework (Additional files 2 and 3). Data were extracted independently by each reviewer and then compared, with disagreement settled through discussion between the two reviewers.

Based on previous categorisations in the literature [7, 17], frameworks were categorised as (1) process models (used to describe and/or guide the process of translating research to practice); (2) determinant frameworks (used to understand determinants that act as barriers and enablers influencing implementation outcomes); (3) classic theories (originating from disciplines external to implementation science and which can be applied to understand or explain aspects of implementation); (4) implementation frameworks (developed by implementation scientists and used to describe and understand features of implementation); (5) evaluation frameworks (used to specify aspects of implementation that could be evaluated to determine implementation success), and (6) scale-up frameworks (used to guide design of processes and factors that support uptake and use at scale) (Additional file 2).

Thematic content analysis was undertaken using a recursive deductive approach in NVivo 12 software [18]. Content analysis allows for replicable and valid inferences from data to their context, with the purposes of providing knowledge, new insights, a representation of facts and a practical guide to action [19]. The combined approach followed three steps, as follows: (1) codebook construction based on published health economic texts [13–15], with openness to new codes arising from the data, (2) coding and cross-coding, and (3) abstraction into themes [20]. One author (VB) coded all frameworks, with two authors (HT, MB) cross-coding four randomly selected frameworks each. Each step of the coding process was discussed and reviewed by three authors (VB, HT, MB), including the abstraction into key themes emerging from the data.

## Results

Twenty-three frameworks were initially included in our analyses, with 15 frameworks included from the paper by Birken et al. [8] and a further 8 frameworks included through cross-referencing against the paper by McKay et al. [17] (Additional file 2). Three frameworks had relatively recently published revisions, which were also included — Reach, Effectiveness, Adoption, Implementation, Maintenance (RE-AIM) [21]; Theoretical Domains Framework (TDF) [22], and the Integrated Promoting Action on Research Implementation in Health Services (iPARiHS) [23]. A total of 26 framework publications, from 23 discrete framework inclusions, were included in the thematic content analysis (Additional file 2).

Five determinant frameworks were included [22–28], along with six process models [3, 29–33], four implementation frameworks [34–37], two evaluation frameworks [21, 38, 39], three classic theories [40–42] and three scale-up frameworks [43–45]. Given that many of the frameworks were developed through literature review or expert opinion (Additional file 2), there were conceptual or content similarities between several frameworks included in our analysis. For instance, Nine Steps for Developing a Scale-Up Strategy [3] and Scaling up Health Service Innovations – A Framework for Action [43] are both WHO resources reporting a conceptual framework of the elements of scale-up. Nine Steps for Developing a Scale-Up Strategy [3] further elaborates on the framework compared to Scaling up Health Service Innovations – A Framework for Action [43] through the incorporation of strategic choice areas and by defining the process for developing a scale-up strategy. The Framework for Scaling Up Physical Activity Interventions [32] was based on RE-AIM [21, 38] and further supplemented by two additional frameworks, the Nine Steps for Developing a Scale-Up Strategy [3] (also separately included in our analysis as it was reported as commonly used [16]) and the Framework for Disseminating Evidence-Based Health Promotion Practices [46] (not included in our analysis as it was not reported as commonly used [8, 17]). The Active Implementation Framework [27] refers to the collective classification of four frameworks (i.e. Implementation Drivers (also separately included in our analysis [26]), Implementation Stages, Policy-Practice Feedback Loops, and Organised Expert Implementation Support).

All included frameworks incorporate economic-related constructs in some form [3, 21–45, 47–49], although there is wide variation in the scope and level of detail of the constructs included between frameworks. Frameworks differ in terms of the extent of inclusion of different economic constructs, the comprehensiveness of inclusion of these constructs and the ways in which they are integrated into the broader frameworks. For example, A Guide to Scaling Up Population Health Interventions [33] incorporates economic constructs, such as costs and resources, in each of the four steps to scaling-up (i.e. (1) scalability assessment, (2) developing a scale-up plan, (3) preparing for scale-up and (4) scaling-up). However, the Theory of Planned Behaviour only briefly touches on economic constructs, for instance, noting that the intention to perform a behaviour is prompted, at least to some degree, by non-motivational factors such as the availability of resources [41] (Additional files 2 and 3). Additional file 3 provides examples of how economic constructs are incorporated, by implementation framework.

**Fig. 1:**
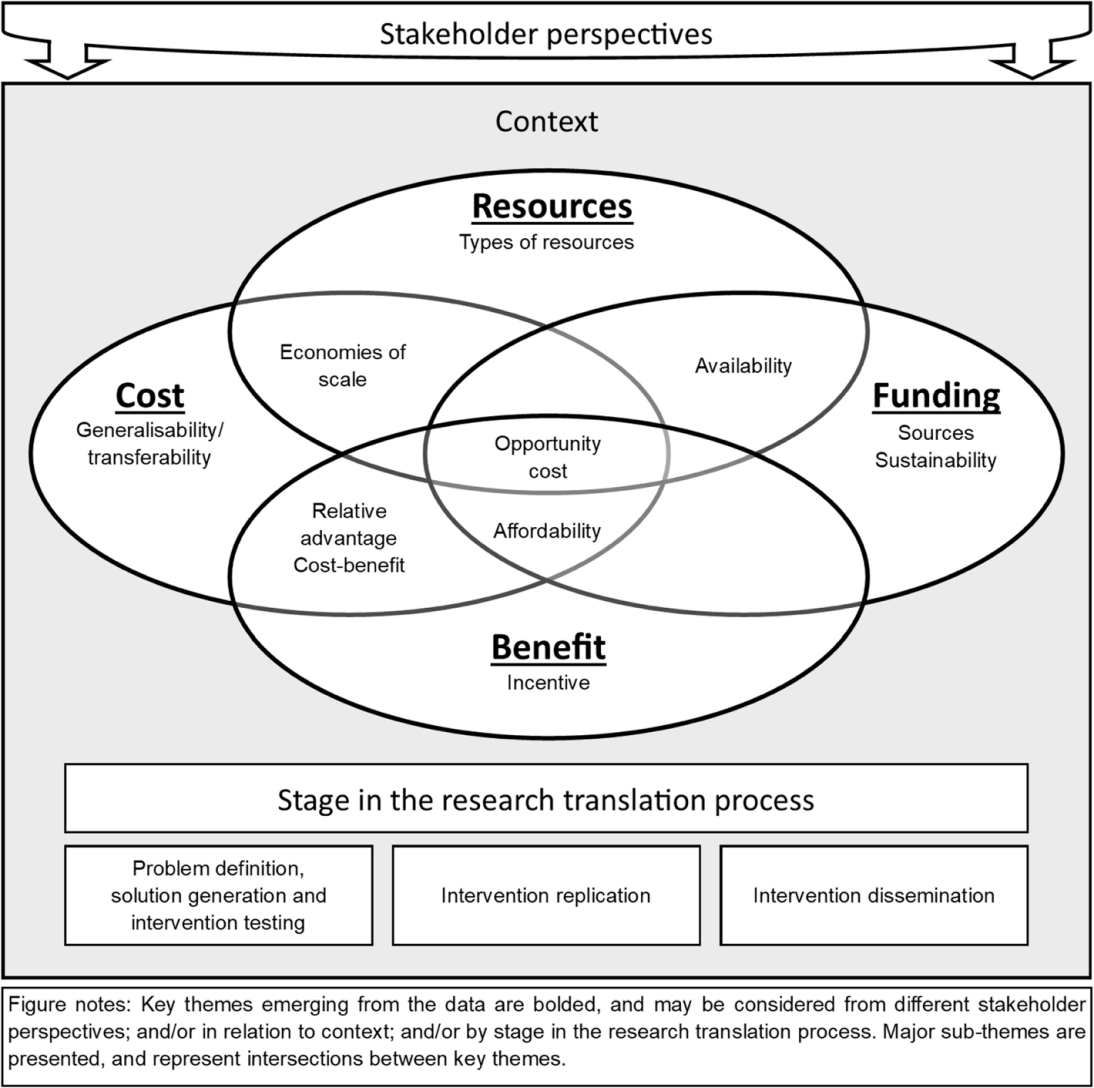
The key themes emerging from the data

Four key themes emerged from the data, related to ‘Resources’, ‘Benefit’, ‘Cost’ and ‘Funding’ (Fig. 1, Table 1). Overarching lenses from which to consider these key themes also emerged, including ‘Stakeholder perspectives’, ‘Context’ and ‘Stage in the research translation process’. The key themes of ‘Resources’, ‘Benefit’, ‘Cost’ and ‘Funding’ may be considered from a specific ‘Stakeholder perspective’ (for example, the perspective of the individual, the organisation or the funder) although, often, the perspective was not explicitly specified or considered. The key themes of ‘Resources’, ‘Benefit’, ‘Cost’ and ‘Funding’ may also be influenced by ‘Context’, either explicitly denoted as the economic context or more broadly considered as the environmental or organisational context. Key themes could also be considered by ‘Stage in the research translation process’ to support evidence-based policy and practice (i.e. (1) problem definition, solution generation and intervention testing, (2) intervention replication and (3) intervention dissemination) [50] although, again, this was not captured in every framework.

**Table 1:** Framework inclusions of key themes emerging from the data

Framework	Benefit	Cost	Resources	Funding
Active implementation frameworks [27]			✓	✓
A guide to scaling up population health interventions [33]	✓	✓	✓	✓
Behaviour change wheel [35]		✓	✓	✓
CFIR [24]	✓	✓	✓	
Diffusion of innovations theory [40]	✓	✓	✓	
Dynamic sustainability framework [37]	✓		✓	
EPIS [29]	✓	✓	✓	✓
Framework for effective implementation [36]	✓	✓	✓	✓
Framework for scaling up physical activity interventions [32]				
Implementation drivers framework [26]			✓	✓
Interactive systems framework for dissemination and implementation [44]			✓	✓
Knowledge to action framework [30]	✓		✓	
Nine steps for developing a scale-up strategy [3]	✓	✓	✓	✓
Normalisation process model [31]		✓	✓	✓
Organisational theory of implementation of innovations [34]	✓		✓	
Proctor’s implementation outcomes [39]		✓	✓	✓
PARiHS [28]				
iPARiHS (revised version) [23]	✓		✓	
RE-AIM [38]	✓	✓	✓	
RE-AIM (revised version) [21]	✓	✓	✓	
Scaling-up: a framework for success [45]	✓		✓	
Scaling up health service innovations – a framework for action [43]	✓	✓	✓	✓
Social cognitive theory [47]	✓	✓		
TDF [25]	✓	✓	✓	
TDF (revised version) [22]	✓		✓	
Theory of planned behaviour [49]	✓	✓	✓	

*CFIR* Consolidated Framework for Implementation Research, *EPIS* Exploration, Preparation, Implementation, Sustainment, *iPARiHS* Integrated Promoting Action on Research Implementation in Health Services, *PARiHS* Promoting Action on Research Implementation in Health Services, *RE-AIM* Reach, Effectiveness, Adoption, Implementation, Maintenance, *TDF* Theoretical Domains Framework

### Resources

The first key theme that emerged from the data related broadly to ‘Resources’ was incorporated into almost 85% of frameworks (22 of 26 included frameworks; Table 1) [3, 21–27, 29–31, 33–41, 43, 44, 49]. ‘Resources’ were recognised as both facilitators and barriers to implementation and scale-up and the ‘Availability’ of resources was a relatively common sub-theme [3, 22, 24, 25, 27, 29, 40]. For example, the Knowledge to Action framework notes that barriers for potential adopters of an intervention may be resource related (e.g. if resources are unavailable or not well-targeted) and should be addressed for successful translation from evidence to practice [30]. The Consolidated Framework for Implementation Research (CFIR) [24] notes that a tangible indicator of organisational readiness for implementation is the availability of resources.

Some frameworks provide explicit detail on the ‘Types’ of resources that may be required for successful implementation or scale-up such as time, equipment, labour, training and education [24, 26, 27, 29, 35]. For example, the consideration of resources, such as labour, time or infrastructure requirements, is a key step for scalability assessment in A Guide to Scaling Up Population Health Interventions [33]. Scaling-Up: A Framework for Success is less explicit in terms of ‘Types’ of resources but notes that some capacity is required to help local decision-makers adopt an intervention [45].

The concept of ‘Opportunity cost’, or the value of the benefit foregone had resources been used elsewhere, is only considered in a small number of frameworks [24, 29, 38]; however, the construct links all four key themes (Fig. 1). The RE-AIM framework [38] notes that even relatively inexpensive interventions can have substantial negative societal effects, including misplaced resources and large opportunity costs when implemented at scale. The sub-theme of ‘Economies of scale’ is also considered in a small number of frameworks [3, 40, 43], linking the key themes of ‘Resources’ and ‘Cost’. ‘Economies of scale’ are generally assessed in the context of cost minimisation, efficiency and the adoption of innovation. For instance, the Nine Steps for Developing a Scale-Up Strategy [3] framework considers whether economies of scale related to cost and resource mobilisation are possible during several steps of the scale-up process.

A small number of frameworks incorporate the concept of ‘Resources’ that may be specifically relevant to different ‘Stages in the research translation process’ [27, 29]. For instance, the Exploration, Preparation, Implementation, Sustainment (EPIS) framework focuses on the proposed four-phase model of the implementation process and notes that resources are required throughout the implementation phase [29]. The potential scarcity of resources for evaluation of implementation and scale-up is also considered in a number of frameworks [21, 33].

The contextual nature of resources (‘Context’) is considered in several but not all frameworks. For instance, the CFIR discusses resources in terms of both the inner and outer setting; the inner setting includes features of structural, political and cultural contexts through which implementation proceeds and the outer setting includes an organisation’s economic, political and social context [24]. The revised version of RE-AIM [21] considers external context, including factors of the external environment such as resources. The TDF includes environmental context and resources as one of the key domains in both the original and the revised versions [22, 25] and the Normalisation Process model considers the distribution of resources within the organisational context [31]. The Dynamic Sustainability Framework considers that context carries its own set of characteristics, including human and capital resources, that are important for implementation [37].

### Benefit

The second theme related to the concept of ‘Benefit’, as broadly incorporated into almost 70% of framework inclusions (18 of 26 included frameworks; Table 1) [3, 21–25, 29, 30, 33, 34, 36–38, 40–43, 45]. ‘Incentivisation’ was a sub-theme of the concept of ‘Benefit’ and mentioned in several frameworks. For instance, the Organisational Theory of Implementation of Innovations notes that incentivisation can foster innovation use [34]. Incentivisation is included in the CFIR as a characteristic of both the inner setting and outer setting framework domains [24]; the implementation climate within the inner setting of an organisation may be strengthened through incentivisation (such as promotions or raises in salaries) and may also act as an external strategy to spread interventions (such as pay-for-performance collaboratives). The Diffusion of Innovations Theory [40] states that the main function of an incentive for adopters is to increase the degree of relative advantage of a new idea.

The sub-theme of ‘Relative advantage’ of implementation or scale-up over existing practice links the key themes of ‘Cost’ and ‘Benefit’ (Fig. 1). For example, both the CFIR [24] and the Nine Steps for Developing a Scale-Up Strategy [3] cite relative advantage (i.e. the advantage of implementing an intervention versus an alternative) as a key intervention characteristic for implementation and scale-up. Relative advantage over existing practices may convince potential users that the costs of implementation are warranted by the benefits [3].

‘Benefits’ are not comprehensively considered from different ‘Stakeholder perspectives’ within the included frameworks, although the CFIR incorporates the concept of relative advantage from the stakeholders’ perspective but does not elaborate on the potential differences in benefit between stakeholder groups [24]. The contextual nature of benefit is acknowledged in the Knowledge to Action process by recognising that decisions about the value and usefulness of knowledge may be influenced by setting or circumstance [30]. Limited consideration of ‘Benefit’ by research stage exists or of how the concept of benefit may change as research moves from evidence to practice (i.e. by ‘Stage in the research translation process’). A Guide to Scaling Up Population Health Interventions [33] includes initially assessing effectiveness at the point of determining suitability for scale-up (step 1) and through ongoing systems for monitoring performance (including effectiveness) at scale-up (step 4).

### Cost

The third theme that emerged from the data was related to ‘Cost’. The ‘Cost’ theme emerged from more than half of the included frameworks (15 of 26 included frameworks) [3, 21, 24, 25, 29, 31, 33, 35, 36, 38–40, 42, 43, 49], with wide heterogeneity between frameworks in terms of frequency of consideration, types of costs and the ways in which cost considerations are incorporated. For instance, cost (and resource) mobilisation is a key strategic choice area featured in Scaling up Health Service Innovations – A Framework for Action [43] and the Nine Steps for Developing a Scale-Up Strategy [3]. Proctor’s Implementation Outcomes [39] includes cost as a distinct implementation outcome. Other frameworks, such as Promoting Action on Research Implementation in Health Services (PARiHS) [28] or the iPARiHS [23], do not include cost constructs at all (Table 1).

Several frameworks consider the ‘Generalisability or transferability’ of ‘Cost’ between settings [21, 39, 43]. For instance, RE-AIM has evolved over time to address issues such as adaptation costs [21]. Adaptation costs may be influenced by ‘Context’. For instance, Proctor’s Implementation Outcomes [39] notes the effect of environmental context on cost. How an intervention relates to the organisational context in which it is set, including decisions about cost, is also included in the Normalisation Process Model [31].

A number of frameworks consider costs in relation to potential benefit [29, 42] or effectiveness (‘Cost-benefit’) [3, 36, 39]. The Guide for Scaling Up Population Health Interventions [33] considers ‘Affordability’, noting that it may be important in gaining support for an intervention from decision-makers. The original version of the TDF [25] specifically framed beliefs about implementation consequences in terms of the benefits versus the costs. Refinement of the framework in 2012 resulted in the addition of two extra domains, yet it provided less detail on each domain, meaning that the explicit cost construct within the framework publication was omitted [22]. In contrast, the original version of RE-AIM [38] was revised to more specifically include cost considerations from ‘Stakeholder perspectives’ [21]. The perspective for cost consideration is also incorporated in the CFIR [24], which highlights the need to minimise costs of implementation to patients specifically (i.e. the patient perspective).

A number of included frameworks consider ‘Cost’ by ‘Stage in the research translation process’ [21, 24, 29, 33]. For example, the EPIS framework [29] considers costs explicitly in relation to four phases of the implementation process (i.e. the exploration, adoption decision, implementation and sustainability phases). The CFIR [24] includes costs associated with both the implementation of the intervention and the intervention itself, noting that, in many contexts, costs are difficult to capture and available resources may have a more direct effect on implementation.

### Funding

The fourth theme that emerged from the data related broadly to the concept of ‘Funding’, but is incorporated in less than half of the included frameworks (11 of 26 included frameworks; Table 1) [3, 26, 27, 29, 31, 33, 35, 36, 39, 43, 44]. ‘Funding’ is considered in relation to funding ‘Sources’ and ‘Availability’, ‘Sustainability’ or contextual impact (‘Context’). ‘Funding’ is linked with ‘Resources’, as there may be a strong correlation between sufficient funding and adequate resourcing for implementation and scale-up. For example, EPIS notes that the funding context is an important consideration and that fiscal resource availability is critical for implementation [29]. The Normalisation Process Model also considers the contextual nature of funding arrangements to promote or inhibit the operationalisation and embedding of complex interventions [31]. The contextual nature of funding influences the sources of funding available for consideration. Implementation and scale-up may use existing funding sources, or new funding streams may be required [33, 43]. The importance of funding across research stages is incorporated into EPIS [29], which notes that a commitment to ongoing funding is required after initial implementation to encourage sustainability. The Active Implementation Frameworks also note that financial sustainability is an important consideration [27].

## Discussion

Recent studies have called for improved theoretical and model-driven approaches to the implementation of public health interventions, with the ultimate aim of increasing the number of evidence-based interventions moving from research to practice [12, 17, 51]. While a relatively large number of implementation and scale-up frameworks currently exist within the literature [8], the results from our thematic analysis of frameworks commonly used by implementation scientists suggest that the inclusion of economic constructs, such as ‘Cost’, ‘Benefit’, ‘Resources’ and ‘Funding’, are currently heterogeneous in terms of scope and level of detail incorporated. Based on our thematic analysis, only five frameworks incorporated all four identified key themes (i.e. ‘Resources’, ‘Benefit’, ‘Cost’, ‘Funding’) [3, 29, 33, 36, 43] (Table 1). Frameworks also varied substantially in the inclusion of lenses through which key themes were viewed (i.e. ‘Stakeholder perspectives’, ‘Context’ and ‘Stage in the research translation process’). However, economic constructs have been recognised as both important barriers and facilitators to successful implementation and scale-up [2, 4, 52]. Previous evidence suggests that information on programme costs and other economic considerations were deemed fundamental to making effective decisions about the appropriateness and feasibility of population-level programme implementation [2]. Our results therefore suggest that a more nuanced and comprehensive approach to the economic constructs related to implementation and scale-up may be required than is currently incorporated into popular implementation and scale-up frameworks.

Our results demonstrate that the contextual nature of key themes, such as ‘Resources’, ‘Cost’, ‘Benefit’ and ‘Funding’, could be more thoroughly considered in many frameworks in order to better inform implementation and scale-up. While it is recognised that capturing ‘Context’ within implementation science is challenging [53], particularly given that context can change rapidly, understanding the impact of context on economic constructs is of vital importance to both implementation scientists and health economists when considering implementation and scale-up in the ‘real world’. This is because public health intervention occurs within complex adaptive systems [54]. Understanding changes in systems and traditional health outcomes is critical in creating interventions that are adaptive and tailorable to specific contexts [55]. Frameworks that better examine the contextual and system impacts on economic constructs, such as ‘Resources’, ‘Cost’ and ‘Benefit’, would further facilitate the creation and implementation of interventions at scale by better preparing researchers and practitioners to address potential economic-related barriers and enablers.

The ‘Stakeholder perspective’ for consideration of economic constructs is also important as perspective plays an integral role in economic evaluation [56]. Economic constructs within the included frameworks in our analysis did not generally consider the spectrum of different stakeholder perspectives that may be relevant to resource allocation decision-making. Consideration of economic constructs from the perspective of relevant stakeholders may better inform decision-makers of the range of potential costs and benefits and — perhaps even more importantly — to whom these costs and benefits may be expected to accrue. Frameworks that consider how important economic constructs may differ between stakeholders’ perspectives, for instance, from the perspective of the funder to the perspective of the service deliverer, are required.

The sub-theme of ‘Opportunity cost’ links all key themes of ‘Resources’, ‘Benefit’, ‘Cost’ and ‘Funding’ but was only incorporated into a small number of frameworks. Only the EPIS framework [29] incorporated all four key themes emerging from the data (‘Resources’, ‘Benefit’, ‘Cost’, ‘Funding’) as well as the sub-theme of 'Opportunity cost'. EPIS considers competing priorities for funding and the opportunity cost of funding targeted services versus other priority areas [29]. ‘Opportunity cost’ is a key concept within health economics [56] and underpins economic evaluation exploring the costs and benefits of an intervention. Implementation and scale-up impacts on the resources or funds that could have been used elsewhere in the health system and on the benefits that could have been gained from an alternate course of action. It is therefore important that opportunity costs be considered at each stage in the research translation process to support and facilitate successful, effective, and cost-effective implementation and scale-up. Accurate and meaningful estimates of opportunity cost as research moves from evidence to practice are required to better inform resource allocation and priority-setting within real-world contexts and environments.

‘Resources’ was the most common theme from our analysis, included in 22 of 26 framework publications [3, 21–27, 29–31, 33–41, 43, 44, 49]. However, the relatively limited consideration of resources by ‘Research translation process stage’ is an area for future work as both resource requirements and availability are likely to differ as research moves from evidence into practice. More thorough planning of resource requirements and availability by research translation process stage could contribute to more successful translation of research to practice. The potential scarcity of resources for on-going evaluation and monitoring is also an issue that should be addressed to facilitate intervention sustainability once implemented at scale.

The included frameworks did not comprehensively consider how the ‘Benefit’ of an intervention may change as research moves into practice (i.e. by ‘Stage in the research translation process’). Recent work by McCrabb et al. [57] estimated a ‘scale-up’ penalty, whereby obesity prevention interventions that had been scaled-up from preceding randomised controlled trials reported effects that were typically 75% lower than reported in the pre-scale-up efficacy trials. This has clear implications for the cost-effectiveness of an intervention once it is implemented at scale and a more rigorous approach to estimating potential changes in effect and then applying these estimates in economic evaluation is required to better inform decision-makers.

Our results suggesting wide variation in the economic constructs incorporated into commonly used frameworks are unsurprising. The included frameworks did not purport to have an economic focus and the aims and purposes of the included frameworks in our analysis vary significantly (Additional file 2); it follows that the inclusion of economic-related constructs also varies. Frameworks have been recognised as useful for describing and understanding the wide range of influences on how effective interventions are implemented and scaled-up [2]. Economic considerations play an important role but are only one of many factors contributing to successful implementation and scale-up. Greenhalgh et al. state that it is not individual factors that make or break an implementation effort but rather the dynamic interaction between them [58]. The type of framework may also influence the scope and degree of detail of included economic constructs. Classic theories, such as the Diffusion of Innovations Theory [40] or Social Cognitive Theory [42, 47], are designed to understand behaviour change at a much more individual level and are less prescriptive as to how to actually implement change itself within organisations or systems [59] than, for instance, process models or implementation frameworks. Since classic theories are reported as commonly used as part of implementation scientists’ research or practice [8, 17], they may therefore provide less scope for the consideration of the key economic themes that we have identified. It should also be noted that frameworks do not purport to be ‘complete’, with many developed based on the current state of the evidence of implementation and scale-up (Additional file 2). For instance, Proctor’s Implementation Outcomes [39] states that the eight implementation outcomes proposed (i.e. acceptability, adoption, appropriateness, feasibility, fidelity, implementation cost, penetration and sustainability) are the most obvious outcomes given the current state of knowledge and that other concepts may emerge as the field of implementation science progresses.

While the key terminology of economic evaluation (e.g. cost-effectiveness analysis, cost-benefit analysis) was only explicitly mentioned in five framework publications [3, 36, 38, 39, 45], the results from our thematic analysis suggest that there is an emerging focus on the economic considerations of implementation and scale-up within commonly used frameworks. The original inception of the RE-AIM framework noted that, whilst economic constructs were not explicitly included, cost and cost-effectiveness were important factors in determining whether a programme is adopted, implemented consistently or maintained [38]. The omission of economic-related constructs was one of the drivers for the revised RE-AIM framework published in 2019, with an increased emphasis on the assessment of costs and the incorporation of costs, benefits and value as overarching issues within the framework [21]. The iPARiHS framework published in 2016 also aimed to more comprehensively include the wider economic context within which implementation occurs [23] as compared to the PARiHS framework [28].

To date, limited health economic evaluations of interventions that have been successfully scaled-up and implemented have been conducted and the majority of economic evidence on the cost-effectiveness of interventions at scale is based on trial or modelled data. However, results from our analysis suggest that economic constructs related to implementation and scale-up are currently under-explored and that there is significant scope for better understanding the economic considerations of implementation and scale-up. This could result in the minimisation of some of these economic-related barriers to successful implementation and scale-up, with significant policy and practice implications in the ‘real world’. Overall, our results suggest that implementation scientists interested in gaining a better understanding of the economic constructs related to successful intervention implementation and scale-up should carefully consider their selection and use of frameworks, ensuring that relevant economic constructs are incorporated. The findings from this study will also inform a larger body of work to produce a guide to more comprehensively inform the economic considerations of intervention implementation and scale-up within health economic evaluations. Findings from our thematic analysis suggest that the proposed guide may also be of use to implementation scientists by providing a more in-depth framework of economic constructs related to effective and successful intervention implementation and scale-up and guidance on measurement and data collection. The proposed guide may provide support to implementation scientists on how existing frameworks can be adapted to more comprehensively include economic constructs.

Our study was strengthened by the use of a pre-defined codebook, independent data extraction by two reviewers (VB, HT) and the cross-coding of a subset of included frameworks and abstraction into key themes by three reviewers (VB, HT, MB). We have attempted to circumvent the lack of clearly defined and agreed use of terminology within the field of implementation science [17] through our consistent use of language throughout this paper. Frameworks may have been misclassified given the lack of clear definitions available in the literature and the fact that terms are often used inter-changeably [59]. However, by taking a thematic approach we were able to look beyond the inconsistencies in the terminology to consider the emergent themes. Finally, inclusion of the large number of implementation frameworks that exist was beyond the scope of this paper. While we have undertaken a framework inclusion process that capitalises on rigorous, peer-reviewed work published within the field [8, 17], relevant implementation theories, models and frameworks may have been omitted from our analysis. Significant scope exists for future analysis of the ways in which economic constructs are incorporated into omitted implementation theories, models and frameworks.

## Conclusions

Effective and cost-effective interventions must be delivered at scale to achieve population health benefits. Implementation and scale-up frameworks enhance the dissemination of research by making the spread of evidence-based interventions more likely [60], by advancing knowledge about which interventions may work best in which contexts and by guiding the development of reliable approaches to ensure successful implementation and scale-up [61]. Our thematic analysis identified significant heterogeneity in the inclusion of economic constructs in commonly used implementation and scale-up frameworks. Given the call for improved theoretical approaches to implementation and scale-up [12] and the evidence that indicates that economic constructs may be both barriers and facilitators to successful scale-up and implementation of evidence-based practices [4, 62], this suggests that a more comprehensive approach to the exploration and consideration of economic constructs related to implementation and scale-up is warranted.

## Supplementary information

**Additional file 1.** Standards for reporting qualitative research

**Additional file 2.** Overview of commonly used theories, models and frameworks

**Additional file 3.** Examples of economic-related factors considered in commonly used implementation frameworks

## Data Availability

All data generated or analysed during this study are available from the corresponding author on reasonable request.

## Abbreviations

CFIR: Consolidated Framework for Implementation Research
EPIS: Exploration, Preparation, Implementation, Sustainment
iPARiHS: Integrated Promoting Action on Research Implementation in Health Services
PARiHS: Promoting Action on Research Implementation in Health Services
RE-AIM: Reach, Effectiveness, Adoption, Implementation, Maintenance
TDF: Theoretical Domains Framework
