# Correction to: a narrative review of economic constructs in commonly used implementation and scale-up theories, frameworks and models

**DOI:** 10.1186/s12961-020-00649-y

**Published:** 2020-10-28

**Authors:** Vicki Brown, Huong Tran, Miranda Blake, Rachel Laws, Marj Moodie

**Affiliations:** 1 Deakin Health Economics, Institute for Health Transformation, Deakin University, Geelong, 3220 Australia; 2 Global Obesity Centre, Institute for Health Transformation, Deakin University, Geelong, 3220 Australia; 3 Institute for Physical Activity and Nutrition, Deakin University, Geelong, 3220 Australia

## Correction to: Health Research Policy and Systems (2020) 18:115 10.1186/s12961-020-00633-6

It was highlighted that in the original article the given and family name of the first author was interchanged and thus tagged incorrectly. This Correction article shows the correct name order. The original article has been updated.

