# Supplementary material for: A narrative review of economic constructs in commonly used implementation and scale-up theories, frameworks and models
Source: Health Res Policy Syst. 2020 Oct 1;18:115. doi: 10.1186/s12961-020-00633-6 (PMC7528255; doi:10.1186/s12961-020-00633-6)
Supplement: Supplementary file 1 — Additional file 1. Standards for reporting qualitative research [file 12961_2020_633_MOESM1_ESM.docx]

**A narrative review of economic constructs in commonly used implementation theories, frameworks and models**

Brown Vicki^1,2^, Tran Huong^1,2^, Blake Miranda^2^, Laws Rachel^3^, Moodie Marj^1,2^

**Additional File 1**

1 Deakin University, Geelong, Australia 3220, Deakin Health Economics, Institute for Health Transformation

2 Deakin University, Geelong, Australia 3220, Global Obesity Centre, Institute for Health Transformation

3 Deakin University, Geelong, Australia 3220, Institute for Physical Activity and Nutrition

Corresponding author: Dr Vicki Brown

Victoria.brown@deakin.edu.au

**Additional File 1 - Standards for Reporting Qualitative Research^1^**

| **No.** | **Topic** | **Page number in manuscript** |
| --- | --- | --- |
| S1 | Title: Concise description of the nature and topic of the study. Identifying the study as qualitative or indicating the approach (e.g., ethnography, grounded theory) or data collection methods (e.g., interview, focus group) is recommended. | Title page |
| S2 | Abstract: Summary of key elements of the study using the abstract format of the intended publication; typically includes background, purpose, methods, results, and conclusions. | Abstract, page 2 |
| S3 | Problem Formulation: Description and significance of the problem/phenomenon studied; review of relevant theory and empirical work; problem statement | Background, pages 4-5 |
| S4 | Purpose or research question: Purpose of the study and specific objectives or questions. | Background, page 5, from line 104  Section 2.1, page 6 |
| S5 | Qualitative approach and research paradigm: Qualitative approach (e.g., ethnography, grounded theory, case study, phenomenology, narrative research) and guiding theory if appropriate; identifying the research paradigm (e.g., post-positivist, constructivist/interpretivist) is also recommended. | Title  Section 2, Methods  Page 8, from line 164 |
| S6 | Researcher characteristics and reflexivity: Researchers’ characteristics that may influence the research, including personal attributes, qualifications/experience, relationship with participants, assumptions, and/or presuppositions; potential or actual interaction between researchers’ characteristics and the research questions, approach, methods, results and/or transferability. | N/A |
| S7 | Context: Setting/site and salient contextual factors; rationale. | N/A |
| S8 | Sampling strategy: How and why research participants, documents, or events were selected; criteria for deciding when no further sampling was necessary (e.g., sampling saturation); rationale. | Section 2.2, pages 6 and 7 |
| S9 | Ethical issues pertaining to human subjects: Documentation of approval by an appropriate ethics review board and participant consent, or explanation for lack thereof; other confidentiality and data security issues. | N/A |
| S10 | Data collection methods: Types of data collected; details of data collection procedures including (as appropriate) start and stop dates of data collection and analysis, iterative process, triangulation of sources/methods, and modification of procedures in response to evolving study findings; rationale. | Section 2, Methods, pages 6-8 |
| S11 | Data collection instruments and technologies: Description of instruments(e.g., interview guides, questionnaires) and devices (e.g., audio recorders) used for data collection; if/how the instrument(s)changed over the course of the study. | Section 2.3, pages 7 and 8 |
| S12 | Units of study: Number and relevant characteristics of participants, documents, or events included in the study; level of participation. | Results, page 8, from line 173 |
| S13 | Data processing: Methods for processing data prior to and during analysis, including transcription, data entry, data management and security, verification of data integrity, data coding and anonymization / de-identificationof excerpts. | Section 2.3, pages 7 and 8 |
| S14 | Data analysis: Process by which inferences, themes, etc. were identified and developed, including the researchers involved in data analysis; usually references a specific paradigm or approach; rationale. | Section 2.3, pages 7 and 8 |
| S15 | Techniques to enhance trustworthiness: Techniques to enhance trustworthiness and credibility of data analysis (e.g., member checking, triangulation, audit trail); rationale. | Section 2.3, pages 7 and 8 |
| S16 | Synthesis and interpretation: Main findings (e.g., interpretations, inferences, and themes); might include development of a theory or model, or integration with prior research or theory. | Section 3 Results, pages 8-15 |
| S17 | Links to empirical data: Evidence (e.g.,quotes, field notes, text excerpts, photographs) to substantiate analytic findings. | Section 3 Results, pages 8-15  Additional File 3 |
| S18 | Integration with prior work, implications, transferability, and contribution(s) to the field: Short summary of main findings, explanation of how findings and conclusions connect to, support, elaborate on, or challenge conclusions of earlier scholarship; discussion of scope of application/generalizability; identification of unique contribution(s) to scholarship in a discipline or field. | Section 4 Discussion, pages 16-20 |
| S19 | Limitations: Trustworthiness and limitations of findings. | Section 4 Discussion, page 20, from line 458 |
| S20 | Conflicts of interest: Potential sources of influence or perceived influence on study conduct and conclusions; how these were managed | Declarations, page 22, line 500 |
| S21 | Funding: Sources of funding and other support; role of funders in data collection, interpretation, and reporting. | Declarations, page 22, line 502 |

**REFERENCES**

1. O’Brien BC, Harris IB, Beckman TJ, Reed DA, Cook DA. Standards for Reporting Qualitative Research: A Synthesis of Recommendations. 2014;89(9):1245-1251.
