# Supplementary material for: A narrative review of economic constructs in commonly used implementation and scale-up theories, frameworks and models
Source: Health Res Policy Syst. 2020 Oct 1;18:115. doi: 10.1186/s12961-020-00633-6 (PMC7528255; doi:10.1186/s12961-020-00633-6)
Supplement: Supplementary file 2 — Additional file 2. Overview of commonly used theories, models and frameworks [file 12961_2020_633_MOESM2_ESM.docx]

**A narrative review of economic constructs in commonly used implementation theories, frameworks and models**

Brown Vicki^1,2^, Tran Huong^1,2^, Blake Miranda^2^, Laws Rachel^3^, Moodie Marj^1,2^

**Additional File 2**

1 Deakin University, Geelong, Australia 3220, Deakin Health Economics, Institute for Health Transformation

2 Deakin University, Geelong, Australia 3220, Global Obesity Centre, Institute for Health Transformation

3 Deakin University, Geelong, Australia 3220, Institute for Physical Activity and Nutrition

Corresponding author: Dr Vicki Brown

[Victoria.brown@deakin.edu.au](mailto:Victoria.brown@deakin.edu.au)

**Additional File 2 - Overview of commonly used theories, models and frameworks**

| **Theory, model or framework** | **Category of theoretical approach (1)** | **Aim** | **Suggested use/s** | **Methods for development** | **Discipline** | **Brief summary** |
| --- | --- | --- | --- | --- | --- | --- |
| Active Implementation Frameworks (2) | Determinant framework | To try and close the research-to-practice gap in early childhood and ensure sustainable program success. | Synthesises four overarching frameworks to assist with understanding, designing, explaining, evaluating implementation. | Synthesized implementation research findings across a range of fields and developed four overarching frameworks, referred to as the Active Implementation Frameworks, based on these findings | Child development, education | Four overarching frameworks: Implementation Drivers (above), Implementation Stages (exploration, installation, initial implementation, full implementation), Policy-Practice Feedback Loops (Plan, Do, Study, Act), Organized Expert Implementation Support. |
| A Guide to Scaling Up Population Health Interventions (3) | Process model | To help policy makers, practitioners, researchers and other decision makers decide on appropriate methodological and practical choices for scaling up, and balance what is desirable with what is feasible. | As a practical guide to assist health policy makers, health practitioners and others responsible for scaling up effective population health interventions. It can also be used by researchers in the design of research studies that are potentially suitable for scaling up, particularly where research–practice collaborations are involved. | The guide was informed by a systematic review of scaling up models and methods, and a two-round Delphi process with a sample of senior policy makers, practitioners and researchers actively involved in scaling up processes. | Public health | The guide is divided into four sections: the ‘scalability assessment’, to determine if an intervention is scalable; the ‘developing a scale up plan’, to develop a practical and workable scaling up plan that can be used to convince stakeholders there is a compelling case for action; ‘preparing for scale up’, aiming to identify ways of securing resources needed for going to scale, operating at scale, and building a foundation of legitimacy and support to sustain the scaling up effort through the implementation stage; and ‘scaling up the intervention’, which involves putting the scale up plan into place. |
| **Theory, model or framework** | **Category of theoretical approach (1)** | **Aim** | **Suggested use/s** | **Methods for development** | **Discipline** | **Brief summary** |
| Behaviour Change Wheel (4) | Implementation framework | To present a framework of behaviour change interventions - characterising interventions and linking them to an analysis of the targeted behaviour. | Intervention design, translation of research into practice | A systematic search of electronic databases and consultation with behaviour change experts were used to identify frameworks of behaviour change interventions. These were evaluated according to three criteria: comprehensiveness, coherence, and a clear link to an overarching model of behaviour. A new framework was developed to meet these criteria. | Psychology | At the centre of the framework is a ‘behaviour system’ involving three essential conditions: capability, opportunity, and motivation (the ‘COM-B system’). This forms the hub of a ‘behaviour change wheel’ (BCW), around which are positioned nine intervention functions aimed at addressing deficits in one or more of these conditions (education, persuasion, incentivisation, coercion, training, restriction, environmental restructuring, modelling, enablement); around this are seven categories of policy that could enable those interventions to occur (communication/marketing, guidelines, fiscal, regulation, legislation, environmental/social planning, service provision). |
| Consolidated Framework for Implementation Research (CFIR) (5) | Determinant framework | To provide an over-arching typology – a list of constructs to promote theory development and verification about what works where and why across multiple contexts. | Guide assessments of implementation context; evaluate implementation progress; help explain findings. Researchers can select constructs from the CFIR that are most relevant for their study setting and use these to guide assessment, evaluation, explanation of findings. | Literature review and snowball sampling | Health services research; implementation science | The CFIR comprises five major domains (the intervention, inner and outer setting, the individuals involved, and the process by which implementation is accomplished). Eight constructs were identified related to the intervention (e.g., evidence strength and quality), four constructs were identified related to outer setting (e.g., patient needs and resources), 12 constructs were identified related to inner setting (e.g., culture, leadership engagement), five constructs were identified related to individual characteristics, and eight constructs were identified related to process (e.g., plan, evaluate, and reflect).  The CFIR provides a list of explicitly defined constructs for which data can be collected for building knowledge about what works where, across multiple settings. |
| **Theory, model or framework** | **Category of theoretical approach (1)** | **Aim** | **Suggested use/s** | **Methods for development** | **Discipline** | **Brief summary** |
| Diffusion of Innovations Theory (6) | Classic theory | To explain how innovations diffuse or spread over time through a population, organisation or social system. | A classic theory that is useful for understanding and explaining the spread of innovation. | Developed by E.M Rogers through a synthesis of published literature, first published in 1962. | Widely applied in many fields. | Five adopter categories are identified (innovators, early adopters, early majority, late majority, laggards). Five factors that influence the adoption of an innovation are identified (relative advantage, compatibility, complexity, trialability, observability). |
| Dynamic Sustainability Framework (DSF) (7) | Implementation framework | To propose a framework that involves: continued learning and problem solving, ongoing adaptation of interventions with a primary focus on fit between interventions and multi-level contexts, and expectations for ongoing improvement as opposed to diminishing outcomes over time. | Useful when considering sustainability, with a focus on ongoing improvement as opposed to diminishing outcomes over time. | Expert knowledge and advice from those in the implementation community, however limited detail on exact method for development is given. | Health services research, implementation science. | The DSF considers the elements of the intervention, the context in which the intervention is delivered and the broader ecological system over time. Each element has constituent components, which may vary. |
| Exploration, Preparation, Implementation, Sustainment (EPIS) (8) | Process model | To propose a multi-level, four phase model of the implementation process (i.e., Exploration, Adoption/Preparation, Implementation, Sustainment), derived from extant literature, and apply it to public sector services. | A framework for considering challenges and opportunities in evidence-based practice implementation in public service sectors. | Derived from the literature, but limited information is given on method for development. | Mental health, child welfare, human services | Conceptual model includes domains of exploration, adoption decision/preparation, active implementation and sustainment. Each domain is considered from both the outer and the inner context. |
| **Theory, model or framework** | **Category of theoretical approach (1)** | **Aim** | **Suggested use/s** | **Methods for development** | **Discipline** | **Brief summary** |
| Framework for Effective Implementation (9) | Implementation framework | To assess the impact of implementation on program outcomes. To identify factors affecting the implementation process. | Useful when considering, designing or evaluating implementation. | Literature review | Child and adolescent health, prevention and health promotion | Implementation is inﬂuenced by variables present in ﬁve categories: innovations, providers, communities, the prevention delivery system (i.e., features related to organizational capacity) and the prevention support system (i.e., training and technical assistance). Under favourable circumstances, variables in all ﬁve categories interact and lead to effective implementation, that is, a process for conducting the intervention as planned. |
| Framework for Scaling Up Physical Activity Interventions (10) | Process model | To provide an overview of factors that could help to increase the ratio of success to failure in scaling up physical activity interventions around the world. | A framework can help ensure that researchers focus on the most critical factors in the scaling-up process, and that policy makers and practitioners understand its staged nature. | Mixed-methods approach, included a traditional systematic literature search complemented by an adapted qualitative Delphi process to obtain comprehensive information on scaled-up interventions worldwide. | Physical activity research, public health | The framework accounts for the importance and relevance of both evidence-based practice (the push interventions developed and tested through research that might be scaled up) and practice-based evidence (the pull real-world practitioner experience to inform intervention approaches) in informing the scaling up of physical activity interventions. |
| Implementation Drivers Framework (11) | Determinant framework | For planning effective implementation supports, assessing progress toward implementation capacity and conducting rigorous research on implementation. | Expert facilitations with a group of implementation team members. A tool to help operationalise best practices for each driver. | Literature review, expert interactions, interviews with program developers, meta-analyses of the literature on leadership and analyses of leadership in education. | Child development, education | Implementation Drivers are the key components of capacity and the functional infrastructure supports that enable a program’s success. The three categories of Implementation Drivers are competency, organisation and leadership. The framework asks participants to rate the drivers currently in place, using an implementation lens. The assessed items help to operationalise best practice for each driver. |
| **Theory, model or framework** | **Category of theoretical approach (1)** | **Aim** | **Suggested use/s** | **Methods for development** | **Discipline** | **Brief summary** |
| Interactive Systems Framework for Dissemination and Implementation (12) | Scale-up framework | To present a framework for understanding the needs, barriers, and resources of the different systems for moving knowledge into practice, as well as a structure for summarising existing research and for illuminating priority areas for new research and action. | The framework is intended to be used by different types of stakeholders (e.g., funders, practitioners, researchers) who can use it to see prevention not only through the lens of their own needs and perspectives, but also as a way to better understand the needs of other stakeholders and systems. | It draws explicitly on the knowledge and expertise of prevention practitioners, funding agencies, and support agencies, as well as that of researchers from the ﬁelds of prevention and dissemination. | Public health, violence prevention | The framework presents three systems: the Prevention Synthesis and Translation System (which distils information about innovations and translates it into user-friendly formats); the Prevention Support System (which provides training, technical assistance or other support to users in the ﬁeld); and the Prevention Delivery System (which implements innovations in the world of practice). |
| Knowledge to Action Framework (KTA) (13) | Process model | A conceptual framework for thinking about the process of moving knowledge into action. | To assist in disseminating scientific research to practitioners, policymakers, patients, and the public. To help knowledge producers and users find their way through the complex, iterative, and organic process of knowledge translation. | Review of the literature of >30 planned action theories, identification of common elements. | Not explicit | The KTA process has two concepts: knowledge creation and action. Each concept is comprised of ideal phases or categories (themselves fluid and permeable). Knowledge creation includes knowledge inquiry, knowledge synthesis, knowledge tools/products, identifying the problem, reviewing and selecting knowledge. Action cycle includes adapting knowledge to local context, assessing barriers to knowledge use, selecting tailored implementation interventions, monitoring knowledge use, evaluating outcomes and sustaining knowledge use (cycles). |
| **Theory, model or framework** | **Category of theoretical approach (1)** | **Aim** | **Suggested use/s** | **Methods for development** | **Discipline** | **Brief summary** |
| Nine Steps for Developing a Scale-Up Strategy (14) | Process model | To develop a resource material for scale-up, to facilitate systematic planning. To outline a concise, step-by-step strategy for scale-up. | Planning, review of scaling up interventions. Intended for program managers and others who plan to scale up successfully tested interventions, but it can also be useful in conducting a review of how scaling up is progressing when already underway. | Based on earlier work, the literature and expert opinion. | Public health | The framework links an innovation to be scaled up with four other elements: a resource team that promotes it; a user organisation expected to adopt the innovation; a strategy to transfer it; and an environment in which the transfer takes place.  Nine steps involved in designing a scaling-up strategy are then discussed (planning actions to increase the scalability of the innovation; increasing the capacity of the user organisation to implement scaling up; assessing the environment and planning actions to increase the potential for scaling-up success; increasing the capacity of the resource team to support scaling up; making strategic choices to support vertical scaling; making strategic choices to support horizontal scaling; determining the role of diversification; planning actions to address spontaneous scaling up; finalizing the scaling-up strategy and identifying next steps). Each step illustrates the type of questions which lead the team to specify recommended actions that become the building blocks of the scaling-up strategy. |
| Normalisation Process Model (15) | Process model | To explain the normalisation of complex interventions that assists in explaining the processes by which complex interventions become routinely embedded. | This model can be used to understand the normalisation potential of new techniques and technologies in healthcare settings. | Re-analysis of data from multiple qualitative studies, leading to sets of analytic propositions. | Health service research, sociology | The model explains the normalisation of complex interventions by reference to four factors demonstrated to promote or inhibit the operationalisation and embedding of complex interventions (interactional workability, relational integration, skill-set workability, and contextual integration). |
| **Theory, model or framework** | **Category of theoretical approach (1)** | **Aim** | **Suggested use/s** | **Methods for development** | **Discipline** | **Brief summary** |
| Organisational Theory of Implementation of Innovations (16) | Implementation framework | To present a model of the determinants of the effectiveness of organisational implementation. | Useful to better understand how to achieve the benefits of an innovation, through successful implementation. | Not defined | Not explicit | Implementation effectiveness is a function of the strength of an organisation’s climate for the implementation of that innovation and the fit of that innovation to targeted users’ values. |
| Proctor's Implementation Outcomes (17) | Evaluation framework | Proposes a heuristic, working ‘‘taxonomy’’ of eight conceptually distinct implementation outcomes—acceptability, adoption, appropriateness, feasibility, ﬁdelity, implementation cost, penetration, and sustainability—along with their nominal deﬁnitions. | Conceptualising and measuring implementation outcomes; treatment and implementation strategies | Working group of implementation researchers, literature search, narrative review. | Community health, mental health | The framework distinguishes between three distinct but interrelated types of outcomes—implementation (acceptability, adoption, appropriateness, costs, feasibility, fidelity, penetration, sustainability), service (efficiency, safety, effectiveness, equity  patient-centeredness, timeliness), and client outcomes (satisfaction, function, symptomatology). |
| Promoting Action on Research Implementation in Health Services (PARiHS) (18)  iPARiHS  (revised version) (19) | Determinant framework | To develop a conceptual framework which represents the interplay and interdependence of many factors inﬂuencing the effective uptake of research evidence into practice. In 2016 a revised version of the framework, called the integrated or i-PARIHS framework, was published. | To inform intervention design, implementation | The conceptual framework emerged from several years of experience, working with clinicians (mostly nurses) in helping them to improve the quality of their care by setting clinical standards, introducing audit and quality improvements, and in changing patient services in several community hospitals in one health authority. | Clinical care, nursing | Successful implementation of evidence into practice is a function of the quality and type of evidence, the characteristics of the setting or context and the way in which the evidence is introduced or facilitated into practice.  There were several reasons for updating the original framework, including that it failed to address key dimensions, including the intended targets for implementation and the wider external context (social, political, economic) in which implementation occurs. |
| **Theory, model or framework** | **Category of theoretical approach (1)** | **Aim** | **Suggested use/s** | **Methods for development** | **Discipline** | **Brief summary** |
| Reach, Efficacy, Adoption, Implementation, Maintenance  (RE-AIM) (20)  RE-AIM  (revised version) (21) | Evaluation framework | A model for evaluating public health interventions. To improve assessment and reporting along the dimensions of the framework. | Evaluation of public health interventions | Builds on work by Abrams and colleagues (22), to expand the impact of an intervention as the product of not only reach and efficacy, but also adoption implementation and maintenance. | RE-AIM has been applied most often in public health and health behaviour change research, but increasingly in more diverse content areas and within clinical, community, and corporate settings. | RE-AIM includes five dimensions: Reach, Efficacy, Adoption, Implementation, Maintenance. Dimensions occur at multiple levels and interact to determine the impact of a program or policy.  The most recent version of RE-AIM includes contextual factors, fit and interactions and overarching issues. This includes increasing emphasis on cost and adaptations to programs and expanding the use of qualitative methods to understand “how” and “why” results came about. |
| Scaling-Up: A Framework for Success (23) | Scale-up framework | To develop a framework for explaining successful scale-up. | A framework for explaining successful scale-up, for planners of scale-up processes to use in thinking about strategies for implementing a new program, policy, or intervention to scale. | Literature review and interviews with thought leaders. | Global health, public health | Divides the scaling up process into six categories: attributes of the specific tool or service being scaled up; attributes of the implementers; the chosen delivery strategy; attributes of the ‘adopting’ community; the socio-political context; and the research context |
| **Theory, model or framework** | **Category of theoretical approach (1)** | **Aim** | **Suggested use/s** | **Methods for development** | **Discipline** | **Brief summary** |
| Scaling up Health Service Innovations - A Framework for Action (24) | Scale-up framework | To present a way of thinking about the scale-up process that identifies its primary components, the choices to be made and the circumstances  that may facilitate or hamper effectiveness and sustainability. | Useful when considering scale-up. | Based on the literature, expert meeting and the experience of the authors. | Reproductive health, public health | The framework links an innovation to be scaled up with four other elements: a resource team that promotes it; a user organisation expected to adopt the innovation; a strategy to transfer it; and an environment in which the transfer takes place. Key attributes facilitate successful expansion of innovations. The key dimensions of the scaling-up strategy are: types, dissemination, organisational choices, cost/resource and mobilisation, monitoring and evaluation. |
| Social Cognitive Theory (25, 26) | Classic theory | This approach addresses the sociostructural determinants of health as well as the personal determinants. Emphasises social influence on behaviour change and explains how people regulate their behaviour. | Behaviour change | Evolved from other theories, including social learning theory | Psychology | This theory posits a multifaceted causal structure in which self-efficacy beliefs operate in concert with cognized goals, outcome expectations, and perceived environmental impediments and facilitators in the regulation of human motivation, action, and well-being. Social cognitive theory distinguishes among three modes of agency: direct personal agency, proxy agency that relies on others to act on one’s behest to secure desired outcomes, and collective agency exercised through socially coordinative and interdependent effort. |
| **Theory, model or framework** | **Category of theoretical approach (1)** | **Aim** | **Suggested use/s** | **Methods for development** | **Discipline** | **Brief summary** |
| Theoretical Domains Framework (TDF)(27)  TDF  (revised version) (28) | Determinant framework | To simplify psychological theory relevant to behaviour change and to make it accessible to those involved in evidence based practice implementation. To identify an agreed set of key theoretical constructs for use in studying the implementation of evidence-based practices. The 2012 study aimed to improve the empirical basis of the TDF. | Useful for researchers working with health service managers towards implementation of evidence-based practice, to encourage successful behavioural change. Domains should be considered when explaining failure of implementation, or when designing interventions to achieve improved implementation. | Expert involvement, consensus approach to development and refinement. | Health psychology | The TDF was originally organised into twelve domains to explain behaviour change (knowledge, skills, social/professional role and identity, beliefs about capabilities, beliefs about consequences, motivation and goals, memory, attention and decision processes, environmental context and resources, social influences, emotion regulation, behavioural regulation, and nature of the behaviour). Domains are presented alongside interview questions which can be used to identify the behaviour change processes likely to be most relevant to implementation of specific evidence-based practices.  Refinement of the framework resulted in 14 domains (knowledge, skills, social/professional role and identity, beliefs about capabilities, optimism, beliefs about consequences, reinforcement, intentions, goals, memory, attention and decision processes, environmental context and resources, social influences, emotions and behavioural regulation). |
| Theory of Planned Behaviour (29) | Classic theory | To predict an individual's intention to engage in a behaviour at a specific time and place. The theory was intended to explain all behaviours over which people have the ability to exert self-control. | To understand behaviours, or to implement interventions that will be effective in changing them, or  to gain substantive information about a behaviour’s determinants. | Built on existing theory, examination of past efforts at using measures of behavioural dispositions to predict behaviour. | Social psychology | Behavioural achievement depends on motivation (intention) and ability (behavioural control). Six constructs collectively represent a person's actual control over the behaviour: attitudes, behavioural intention, subjective norms, social norms, perceived power, perceived behavioural control. |

**REFERENCES**

1. Birken SA, Powell BJ, Shea CM, Haines ER, Alexis Kirk M, Leeman J, et al. Criteria for selecting implementation science theories and frameworks: results from an international survey. Implementation Science. 2017;12(1):124.

2. Metz A, Bartley L. Active Implementation Frameworks for Program Success. Chapel Hill: National Implementation Research Network 2012.

3. Milat AJ, Newson R, King L, Rissel C, Wolfenden L, Bauman A, et al. A guide to scaling up population health interventions. Public Health Res Pract. 2016;26(1):e2611604.

4. Michie S, Van Stralen MM, West R. The behaviour change wheel: a new method for characterising and designing behaviour change interventions. Implementation Science. 2011;6(1):42.

5. Damschroder LJ, Aron DC, Keith RE, Kirsh SR, Alexander JA, Lowery JC. Fostering implementation of health services research findings into practice: a consolidated framework for advancing implementation science. Implementation Science. 2009;4(1):50.

6. Rogers EM. Diffusion of Innovations. 3rd edn ed. New York: The Free Press; 1983.

7. Chambers DA, Glasgow RE, Stange KC. The dynamic sustainability framework: addressing the paradox of sustainment amid ongoing change. Implementation Science. 2013;8(1):117.

8. Aarons GA, Hurlburt M, Horwitz SM. Advancing a Conceptual Model of Evidence-Based Practice Implementation in Public Service Sectors. Administration and Policy in Mental Health and Mental Health Services Research. 2011;38(1):4-23.

9. Durlak JA, DuPre EP. Implementation matters: A review of research on the influence of implementation on program outcomes and the factors affecting implementation. American Journal of Community Psychology. 2008;41(3-4):327.

10. Reis RS, Salvo D, Ogilvie D, Lambert EV, Goenka S, Brownson RC. Scaling up physical activity interventions worldwide: stepping up to larger and smarter approaches to get people moving. The Lancet. 2016;388(10051):1337-48.

11. National Implementation Science Network. Implementation Drivers: Assessing Best Practices. Chapel Hill: Unviersity of North Carolina; 2015.

12. Wandersman A, Duffy J, Flaspohler P, Noonan R, Lubell K, Stillman L, et al. Bridging the Gap Between Prevention Research and Practice: The Interactive Systems Framework for Dissemination and Implementation. American Journal of Community Psychology. 2008;41(3-4):171-81.

13. Graham ID, Logan J, Harrison MB, Straus SE, Tetroe J, Caswell W, et al. Lost in knowledge translation: time for a map? Journal of Continuing Education in the Health Professions. 2006;26(1):13-24.

14. World Health Organisation, ExpandNet. Nine steps for developing a scale-up strategy. Geneva, Switzerland: WHO; 2010.

15. May C. A rational model for assessing and evaluating complex interventions in health care. BMC Health Services Research. 2006;6(1):86.

16. Klein KJ, Sorra JS. The Challenge of Innovation Implementation. Academy of Management Review. 1996;21(4):1055-80.

17. Proctor E, Silmere H, Raghavan R, Hovmand P, Aarons G, Bunger A, et al. Outcomes for implementation research: conceptual distinctions, measurement challenges, and research agenda. Administration and Policy in Mental Health. 2011;38(2):65-76.

18. Kitson A, Harvey G, McCormack B. Enabling the implementation of evidence based practice: a conceptual framework. Quality in Health Care. 1998;7(3):149.

19. Harvey G, Kitson A. PARIHS revisited: from heuristic to integrated framework for the successful implementation of knowledge into practice. Implementation Science. 2016;11(1):33.

20. Glasgow RE, Vogt TM, Boles SM. Evaluating the public health impact of health promotion interventions: the RE-AIM framework. American Journal of Public Health. 1999;89(9):1322-7.

21. Glasgow RE, Harden SM, Gaglio B, Rabin B, Smith ML, Porter GC, et al. RE-AIM Planning and Evaluation Framework: Adapting to New Science and Practice With a 20-Year Review. Frontiers in Public Health. 2019;7(64).

22. Abrams DB, Orleans CT, Niaura RS, Goldstein MG, Prochaska JO, Velicer W. Integrating individual and public health perspectives for treatment of tobacco dependence under managed health care: a combined stepped-care and matching model. Annals of Behavioral Medicine. 1996;18(4):290-304.

23. Yamey G. Scaling Up Global Health Interventions: A Proposed Framework for Success. PLoS Med. 2011;8(6):e1001049.

24. Simmons R, Shiffman J. Scaling-up health service innovations - a framework for action. In: Fajans P, Ghiron L, Simmons R, editors. Scaling up health service delivery : from pilot innovations to policies and programmes. Geneva: World Health Organization; 2007.

25. Bandura A. Health promotion for the perspective of social cognitive theory. Psychology and Health. 1998;13:623-49.

26. Bandura A. Social Foundations of Thought and Action. New Jersey: Prentice Hall; 1986.

27. Michie S, Johnston M, Abraham C, Lawton R, Parker D, Walker A. Making psychological theory useful for implementing evidence based practice: a consensus approach. Quality and Safety in Health Care. 2005;14(1):26.

28. Cane J, O’Connor D, Michie S. Validation of the theoretical domains framework for use in behaviour change and implementation research. Implementation Science. 2012;7(1):37.

29. Ajzen I. The theory of planned behavior. Organizational Behavior and Human Decision Processes. 1991;50(2):179-211.
