# Supplementary material for: A narrative review of economic constructs in commonly used implementation and scale-up theories, frameworks and models
Source: Health Res Policy Syst. 2020 Oct 1;18:115. doi: 10.1186/s12961-020-00633-6 (PMC7528255; doi:10.1186/s12961-020-00633-6)
Supplement: Supplementary file 3 — Additional file 3. Examples of economic-related factors considered in commonly used implementation frameworks [file 12961_2020_633_MOESM3_ESM.docx]

**A narrative review of economic constructs in commonly used implementation theories, frameworks and models**

Brown Vicki^1,2^, Tran Huong^1,2^, Blake Miranda^2^, Laws Rachel^3^, Moodie Marj^1,2^

**Additional File 3**

1 Deakin University, Geelong, Australia 3220, Deakin Health Economics, Institute for Health Transformation

2 Deakin University, Geelong, Australia 3220, Global Obesity Centre, Institute for Health Transformation

3 Deakin University, Geelong, Australia 3220, Institute for Physical Activity and Nutrition

Corresponding author: Dr Vicki Brown

[Victoria.brown@deakin.edu.au](mailto:Victoria.brown@deakin.edu.au)

**Additional File 3 – Examples of economic-related constructs considered in commonly used implementation frameworks**

| **Theory, model or framework** | **Examples of economic constructs from the published text** | **Examples of themes (sub-themes) and/or overarching lens from which to view the themes (sub-themes) emerging from the text** |
| --- | --- | --- |
| Active Implementation Frameworks (1) | - The Implementation Stages framework: Installation Stage: Ensuring that financial and human resources are in place, finding physical space or purchasing equipment or technology. - Full Implementation Stage: The time it takes to move from initial implementation to full implementation will vary depending on the complexity of the new program model, the baseline infrastructure, the availability of implementation supports and resources, and other contextual factors. | - Stage in the research translation process, Resources (Availability, Types of resources) - Context, Resources (Availability) |
| A Guide to Scaling Up Population Health Interventions (2) | - Step 1: Scalability assessment. Assess acceptability and feasibility. Judge whether the intervention could realistically be scaled-up, given what is known about its costs, workforce requirements, time required, infrastructure requirements and acceptability to stakeholders. - Step 3: Prepare for scale-up: secure resources and build a foundation of legitimacy for the scaling-up plan. Realign and mobilise resources. Mobilise financial resources through existing channels or through new funding streams. | - Stage in the research translation process, Stakeholder perspective, Cost, Resources (Types of resources) - Stage in the research translation process, Resources, Funding |
| Behaviour Change Wheel (3) | - Interventions: Incentivisation, creating expectation of reward - Policies: Fiscal, using the tax system to reduce or increase the financial cost | - Benefit (Incentive) - Cost |
| Consolidated Framework for Implementation Research (CFIR) (4) | - Relative advantage: Stakeholders’ perceptions of the advantage of implementing the intervention versus an alternative solution… - Costs of the intervention and costs associated with implementing the intervention, including investment, supply and opportunity costs. - Organisation rewards and incentives, including promotions and raises in salary - Available resources: the level of resources dedicated for implementation and ongoing operations including money, training, education, physical space and time. | - Cost, Benefit (Relative advantage) - Cost (Opportunity cost) - Benefit (Incentive) - Resources (Availability, Types of resources) |
| Diffusion of Innovations Theory (5) | - Economic factors and rate of adoption: The initial cost of an innovation may affect its rate of adoption. - “Ups” possess greater slack resources for adopting innovations than “downs”. “Ups” can usually adopt innovations much more easily than “downs”, particularly if these new ideas are expensive and technologically complex, and if they provide economies of scale. | - Cost (Affordability) - Resources (Availability, Economies of scale) |
| **Theory, model or framework** | **Examples of economic constructs included** | **Examples of themes (sub-themes) and/or overarching lens from which to view the themes (sub-themes) emerging from the text** |
| Dynamic Sustainability Framework (DSF) (6) | - The context: The DSF anchors the ultimate benefit of the intervention in terms of its ability to fit within a practice setting, typically a clinical or community setting. This context carries its own set of characteristics, including human and capital resources, information systems, organisational culture, climate and structure, and processes for training and supervision of staff. - Incentives are needed to support ongoing adaptation of interventions, particularly where evidence is limited, specifically including monitoring of progress and documentation of adaptations, using quality measures relevant to stakeholders and patients. | - Context, Resources (Types of resources) - Stakeholder perspectives, Benefit (Incentive) |
| Exploration, Preparation, Implementation, Sustainment (EPIS) (7) | - Exploration phase: The outer context: Sociopolitical and funding contexts influence both the exploration phase and the adoption decision/preparation phase. - Active Implementation Phase: Outer Context: Sociopolitical and funding contexts. Whether an evidence-based practice is part of new services, being integrated into existing services, or replacing existing funded services, fiscal resource availability is critical. The costs of policy and contracting changes involve resources to support staff in leading or attending internal and external meetings, developing requests for proposals, and facilitating proposal review. Funding is necessary for staff training, computer systems, and other materials (e.g., binders, notebooks, manuals, DVDs, training tapes, etc.). Funds for targeted services may face competing priorities of legislatures that may favour funding to cover other increasing costs such as Medicaid and prisons. | - Context, Stage in the research translation process, Funding - Context, Stage in the research translation process, Funding (Sources, Opportunity cost) |
| Framework for Effective Implementation (8) | - Community level factors: Funding. Funding is a necessary but insufﬁcient condition for effective implementation, although many funders do not provide sufﬁcient time or money for implementation. - Prevention support system: Approaches to insure provider proﬁciencies in the skills necessary to conduct the intervention and to enhance providers’ sense of self-efﬁcacy. The combination of resources offered to providers once implementation begins, including retraining in certain skills, training of new staff, emotional support, and mechanisms to promote local problem solving efforts. Ideally, training and technical assistance occur after necessary resources related to time, staff, administrative, and ﬁnancial support have been secured, and other factors are positively disposed toward implementation (shared vision, shared decision-making, effective leadership and support, and so on). | - Funding - Resources (Types of resources, Availability), Funding (Availability) |
| **Theory, model or framework** | **Examples of economic constructs included** | **Examples of themes (sub-themes) and/or overarching lens from which to view the themes (sub-themes) emerging from the text** |
| Framework for Scaling Up Physical Activity Interventions (9) | - This publication doesn’t go into detail about the framework components, but highlights contextual variables, including economic conditions, in the published diagram. | - Context |
| Implementation Drivers Framework (10) | - Systems intervention: Leaders and managers engage with the larger service delivery and funding systems to create improved regulatory and funding environment | - Context, Stage in the research translation process, Funding |
| Interactive Systems Framework for Dissemination and Implementation (11) | - The Prevention Delivery System highlights organisation and community-level factors, such as resources, and the way they interact in complex ways and also interact with the characteristics of an innovation to be implemented. - Dissemination and implementation take place within a broader context not fully captured by the systems identiﬁed within the Framework. Contextual factors include funding and macro-policy. | - Context, Resources - Context, Funding |
| Knowledge to Action Framework (12) | - Barriers for potential adopters may be resource-related, and should be addressed in the action cycle. When the barriers are related more to the organisation of service delivery, introducing reminder systems, modifying the documentation system, changing staffing levels, purchasing equipment, or altering the remuneration process may be useful strategies. | - Resources (Availability, Types of resources) |
| Nine Steps for Developing a Scale-Up Strategy (13) | - Step 1. Planning actions to increase the scalability of the innovation: Relative advantage attribute: Is it more cost-effective than existing practices or alternatives? Establishing costs and assess cost-effectiveness. Relative advantage over existing practices so that potential users are convinced the costs of implementation are warranted by the benefits - Step 2. Increasing the capacity of the user organisation to implement scaling up. Implementation capacity attribute: Does the user organisation have capacity in training, physical facilities and equipment, human resources. Identify opportunities for mobilizing/ sharing resources within the user organization. - Step 3. Assessing the environment and planning actions to increase the potential for scaling-up success. Begin by identifying the various environmental sectors of relevance for scaling up: Consider the policy context and political system, the availability of donor support, etc. - Step 4. Increasing the capacity of the resource team to support scaling up. Resource teams to promote and facilitate wider use of the innovation. A variety of special technical, managerial, leadership and financial inputs are needed to support scaling up. Helping ensure that these are available is a key task of the resource team, which may or may not be officially appointed to act in this role. | - Stage in the research translation process, Cost, Benefit (Relative advantage) - Stage in the research translation process, Resources (Availability) - Stage in the research translation process, Context, Funding (Sources, Availability) - Stage in the research translation process, Resources (Type of resources, Availability) |
| **Theory, model or framework** | **Examples of economic constructs included** | **Examples of themes (sub-themes) and/or overarching lens from which to view the themes (sub-themes) emerging from the text** |
| Normalisation Process Model (14) | - Skill-set workability: How is the current division of labour affected by a complex intervention? - Contextual integration: Execution, includes funding arrangements, decisions about the distribution of resources, costs and risks within the organisation | - Resources (Types of resources) - Context, Funding, Resources, Cost |
| Organisational Theory of Implementation of Innovations (15) | - Although an innovation is extremely unlikely to yield significant benefits to an adopting organization unless the innovation is used consistently and well, effective implementation does not guarantee that the innovation will, in fact, prove beneficial for the organization. | - Stakeholder perspectives, Benefit |
| Proctor's Implementation Outcomes (16) | - Cost (incremental or implementation cost) is defined as the cost impact of an implementation effort. Implementation costs vary according to three components. First, because treatments vary widely in their complexity, the costs of delivering them will also vary. Second, the costs of implementation will vary depending upon the complexity of the particular implementation strategy used. Finally, because treatments are delivered in settings of varying complexity and overheads (ranging from a solo practitioner’s office to a tertiary care facility), the overall costs of delivery will vary by the setting. | - Stage in the research translation process, Context, Cost (Generalisability) |
| Promoting Action on Research Implementation in Health Services (PARiHS) (17) | - Context is included as a framework element. | - Context |
| Integrated PARiHS  (iPARiHS) (18)  *(revised version)* | - Innovation: Relative advantage - Recipients: Time, resources, support | - Benefit (Relative advantage) - Stakeholder perspectives, Resources (Types of resources) |
| Reach, Efficacy, Adoption, Implementation, Maintenance  (RE-AIM)(19) | - Even services that cost only a few dollars can have substantial negative (as well as positive) societal effects, including misplaced resources and large opportunity costs, when delivered to millions of people. | - Cost (Opportunity cost), Benefit |
| RE-AIM (20)  *(revised version)* | - Increased emphasis on the assessment of costs (including implementation, adaptation and dissemination costs), from the perspective of multiple stakeholders and across the various RE-AIM dimensions. Costs, benefits and value are now incorporated as overarching issues. - RE-AIM cost assessments have focused on the multilevel nature of implementation, diﬀerent stakeholder perspectives, and cost estimates for replicating a program or policy in diﬀerent settings. | - Cost, Benefit, Stakeholder perspectives - Cost (Generalisability/transferability), Stakeholder perspectives |
| **Theory, model or framework** | **Examples of economic constructs included** | **Examples of themes (sub-themes) and/or overarching lens from which to view the themes (sub-themes) emerging from the text** |
| Scaling-Up: A Framework for Success (21) | - The framework divides the scaling up process into six categories: attributes of the specific tool or service being scaled-up; attributes of the implementers; the chosen delivery strategy; attributes of the ‘adopting’ community; the socio-political context; and the research context. | - Context |
| Scaling up Health Service Innovations - A Framework for Action (22) | - The environment: The social, cultural, political and economic context within which scaling up takes place (from the model). These include the policy setting, the political system, bureaucratic culture, the health sector, the socioeconomic and cultural contexts and the influence of global trends. - Attributes of the innovation: Relative advantage over existing practices. - The scale-up strategy: Assessing costs and mobilising resources: Costs arise for all types of scaling up: 1) for expanding the innovation to new geographical sites or population groups; 2) for the often considerable time and effort needed to obtain political support and for advocacy to institutionalise the innovation; 3) for diversifying the innovation through the additional testing and implementation of new components; and 4) for evaluating and working with spontaneous scaling up that may arise. | - Context - Cost, Benefit (Relative advantage) - Stage in the research translation process, Cost (Generalisability) |
| Social Cognitive Theory (23) | - Incentive motivators, including monetary incentives, may influence actions. - Proponents of the stage view linked stages to sociocognitive determinants. They showed that self-regulatory efficacy and the balance of expected costs and benefits of change differentiate individuals cast into the various stages. | - Benefit (Incentive) - Stage in the research translation process, Cost, Benefit |
| Theoretical Domains Framework (TDF)(24) | - Environmental context and resources: To what extent do physical or resource factors facilitate or hinder x? - Environmental context and resources: Are there competing tasks and time constraints? | - Context, Resources - Context, Resources (Opportunity cost) |
| TDF (25)  *(revised version)* | - Reinforcement: rewards, incentives, sanctions - Environmental context and resources: resources/material resources | - Benefit (Incentive) - Context, Resources |
| Theory of Planned Behaviour (26) | - The intention to perform a behaviour is motivated at least some degree on such non-motivational factors as availability of requisite opportunities and resources (e.g. time, money etc.). - Costs are included as an attribute of behavioural belief linking the behaviour to a certain outcome. Benefits are linked to outcomes. | - Resources (Availability, Types of resources) - Cost, Benefit |

**REFERENCES**

1. Metz A, Bartley L. Active Implementation Frameworks for Program Success. Chapel Hill: National Implementation Research Network 2012.

2. Milat AJ, Newson R, King L, Rissel C, Wolfenden L, Bauman A, et al. A guide to scaling up population health interventions. Public Health Res Pract. 2016;26(1):e2611604.

3. Michie S, Van Stralen MM, West R. The behaviour change wheel: a new method for characterising and designing behaviour change interventions. Implementation Science. 2011;6(1):42.

4. Damschroder LJ, Aron DC, Keith RE, Kirsh SR, Alexander JA, Lowery JC. Fostering implementation of health services research findings into practice: a consolidated framework for advancing implementation science. Implementation Science. 2009;4(1):50.

5. Rogers EM. Diffusion of Innovations. 3rd edn ed. New York: The Free Press; 1983.

6. Chambers DA, Glasgow RE, Stange KC. The dynamic sustainability framework: addressing the paradox of sustainment amid ongoing change. Implementation Science. 2013;8(1):117.

7. Aarons GA, Hurlburt M, Horwitz SM. Advancing a Conceptual Model of Evidence-Based Practice Implementation in Public Service Sectors. Administration and Policy in Mental Health and Mental Health Services Research. 2011;38(1):4-23.

8. Durlak JA, DuPre EP. Implementation matters: A review of research on the influence of implementation on program outcomes and the factors affecting implementation. American Journal of Community Psychology. 2008;41(3-4):327.

9. Reis RS, Salvo D, Ogilvie D, Lambert EV, Goenka S, Brownson RC. Scaling up physical activity interventions worldwide: stepping up to larger and smarter approaches to get people moving. The Lancet. 2016;388(10051):1337-48.

10. National Implementation Science Network. Implementation Drivers: Assessing Best Practices. Chapel Hill: Unviersity of North Carolina; 2015.

11. Wandersman A, Duffy J, Flaspohler P, Noonan R, Lubell K, Stillman L, et al. Bridging the Gap Between Prevention Research and Practice: The Interactive Systems Framework for Dissemination and Implementation. American Journal of Community Psychology. 2008;41(3-4):171-81.

12. Graham ID, Logan J, Harrison MB, Straus SE, Tetroe J, Caswell W, et al. Lost in knowledge translation: time for a map? Journal of Continuing Education in the Health Professions. 2006;26(1):13-24.

13. World Health Organisation, ExpandNet. Nine steps for developing a scale-up strategy. Geneva, Switzerland: WHO; 2010.

14. May C. A rational model for assessing and evaluating complex interventions in health care. BMC Health Services Research. 2006;6(1):86.

15. Klein KJ, Sorra JS. The Challenge of Innovation Implementation. Academy of Management Review. 1996;21(4):1055-80.

16. Proctor E, Silmere H, Raghavan R, Hovmand P, Aarons G, Bunger A, et al. Outcomes for implementation research: conceptual distinctions, measurement challenges, and research agenda. Administration and Policy in Mental Health. 2011;38(2):65-76.

17. Kitson A, Harvey G, McCormack B. Enabling the implementation of evidence based practice: a conceptual framework. Quality in Health Care. 1998;7(3):149.

18. Harvey G, Kitson A. PARIHS revisited: from heuristic to integrated framework for the successful implementation of knowledge into practice. Implementation Science. 2016;11(1):33.

19. Glasgow RE, Vogt TM, Boles SM. Evaluating the public health impact of health promotion interventions: the RE-AIM framework. American Journal of Public Health. 1999;89(9):1322-7.

20. Glasgow RE, Harden SM, Gaglio B, Rabin B, Smith ML, Porter GC, et al. RE-AIM Planning and Evaluation Framework: Adapting to New Science and Practice With a 20-Year Review. Frontiers in Public Health. 2019;7(64).

21. Yamey G. Scaling Up Global Health Interventions: A Proposed Framework for Success. PLoS Med. 2011;8(6):e1001049.

22. Simmons R, Shiffman J. Scaling-up health service innovations - a framework for action. In: Fajans P, Ghiron L, Simmons R, editors. Scaling up health service delivery : from pilot innovations to policies and programmes. Geneva: World Health Organization; 2007.

23. Bandura A. Health promotion for the perspective of social cognitive theory. Psychology and Health. 1998;13:623-49.

24. Michie S, Johnston M, Abraham C, Lawton R, Parker D, Walker A. Making psychological theory useful for implementing evidence based practice: a consensus approach. Quality and Safety in Health Care. 2005;14(1):26.

25. Cane J, O’Connor D, Michie S. Validation of the theoretical domains framework for use in behaviour change and implementation research. Implementation Science. 2012;7(1):37.

26. Ajzen I. The theory of planned behavior. Organizational Behavior and Human Decision Processes. 1991;50(2):179-211.
